# Supplementary material for: Integration of Metabolomic and Transcriptomic Analyses Reveals the Molecular Mechanisms of Flower Color Formation in Prunus mume
Source: Plants (Basel). 2024 Apr 11;13(8):1077. doi: 10.3390/plants13081077 (PMC11054544; doi:10.3390/plants13081077)
Supplement: Supplementary file 1 [file plants-13-01077-s001.zip › Supplementary Figures S1-S8.pdf]

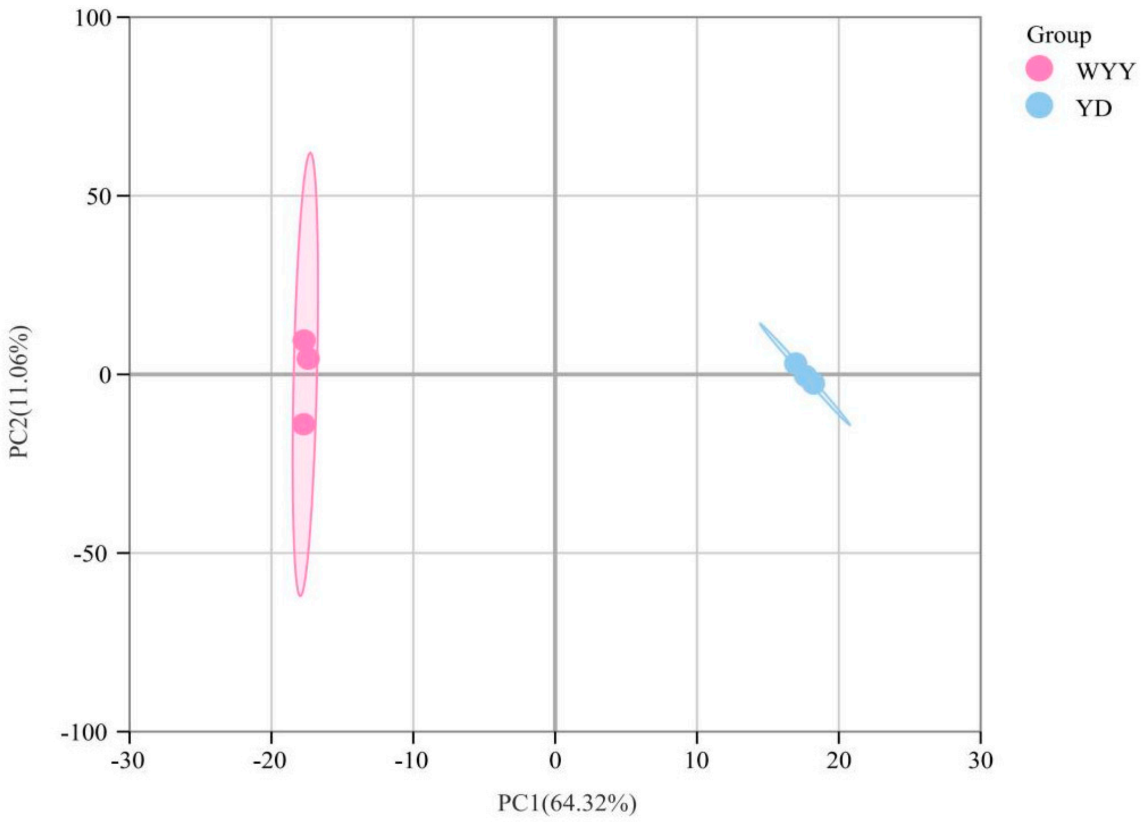

Supplemental Figure S1. Principal component analysis of LC-MS. Blue represents white flower variety YD, red represents purple-red variety WYY

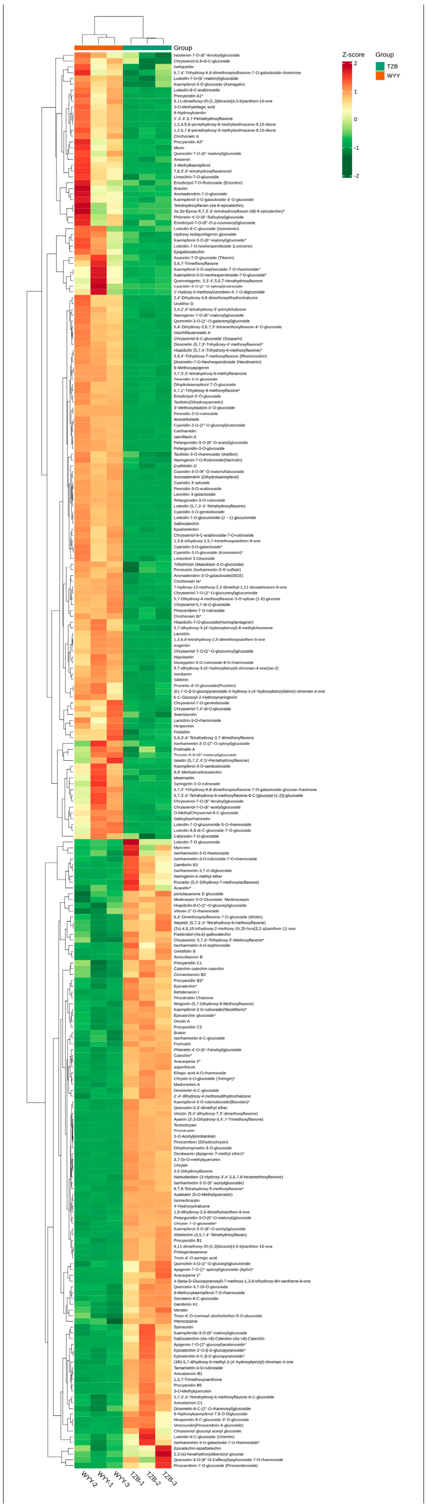

Supplemental Figure S2. Clustering heat map of all differentially accumulated metabolites. Green represents the white variety YD and red represents the purple-red variety WYY. Horizontal is the sample name, vertical is the metabolite information, Group is the grouping, different colors are the colors filled with different values obtained by standardized treatment of different relative contents (red represents high content, green represents low).

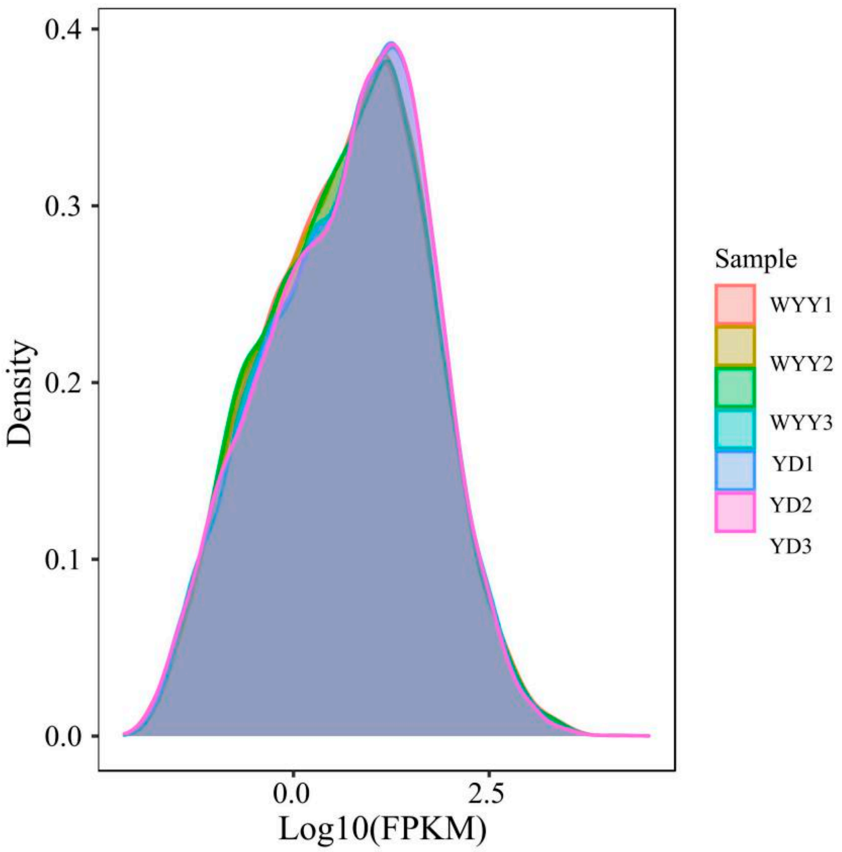

Supplemental Figure S3. mRNA expression distribution of 6 samples from 2 *P. mume* cultivars. The curves of different colors represent different samples, the Abscissa of the point on the curve represents the logarithm of the corresponding sample FPKM, and the ordinate of the point represents the probability density.

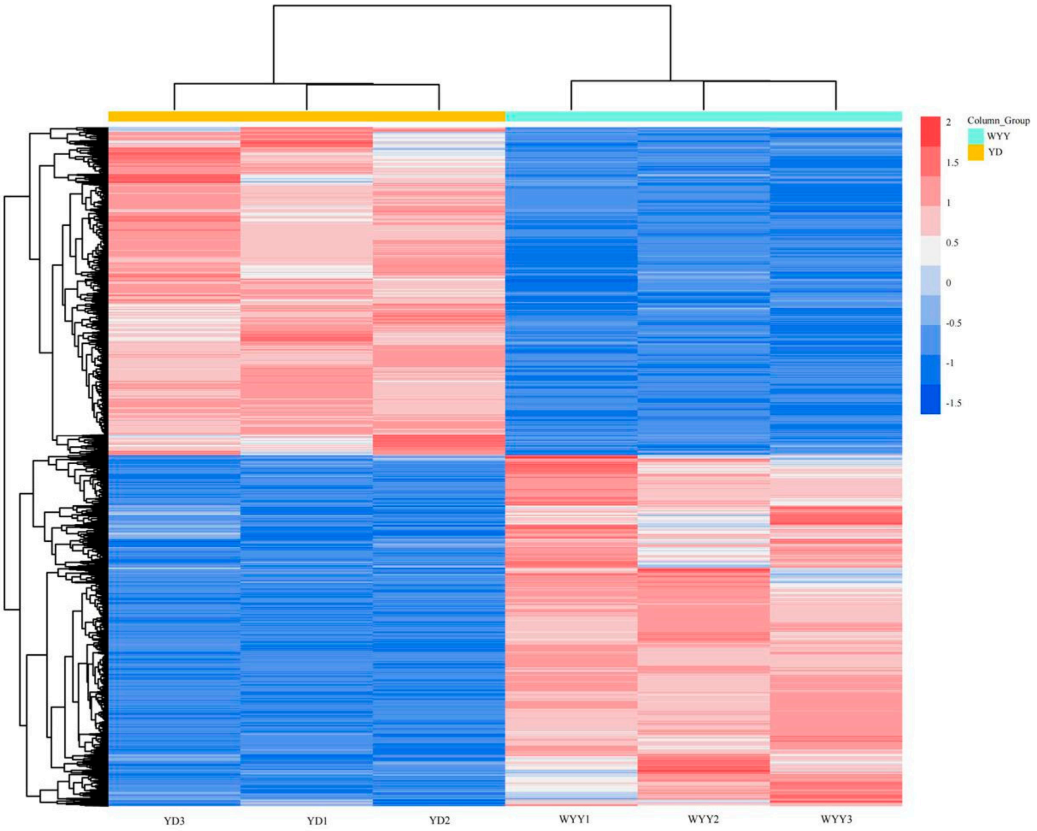

Supplemental Figure S4. DEGs heatmap of Pearson correlation coefficient analysis showing the correlation between the expression levels of pairwise samples. Abscissa denotes sample name and hierarchical clustering result; ordinate indicates differential gene and hierarchical clustering result. Red indicates high expression and blue indicates low expression.

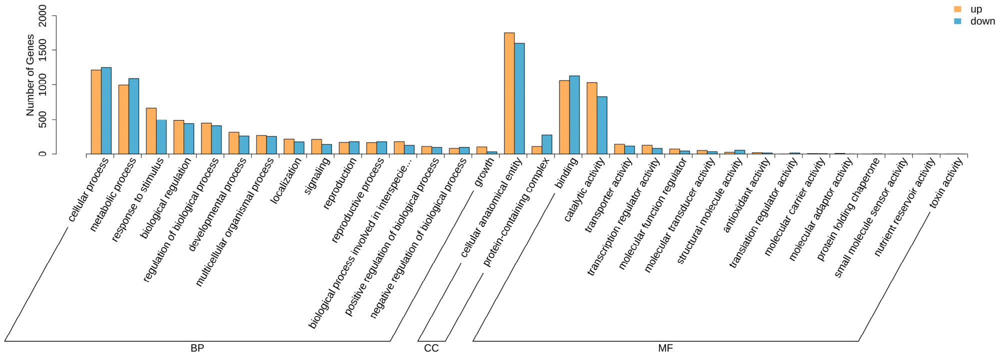

Supplemental Figure S5. GO enrichment analysis of DEGs in two *P. mume* cultivars. Abscissa denotes secondary GO entries, and ordinate indicates the number of differential genes in GO entries. BP is Biological process, CC is Cellular component, MF is Molecular function. The ordinate is the number of differentially expressed genes annotated to this item.

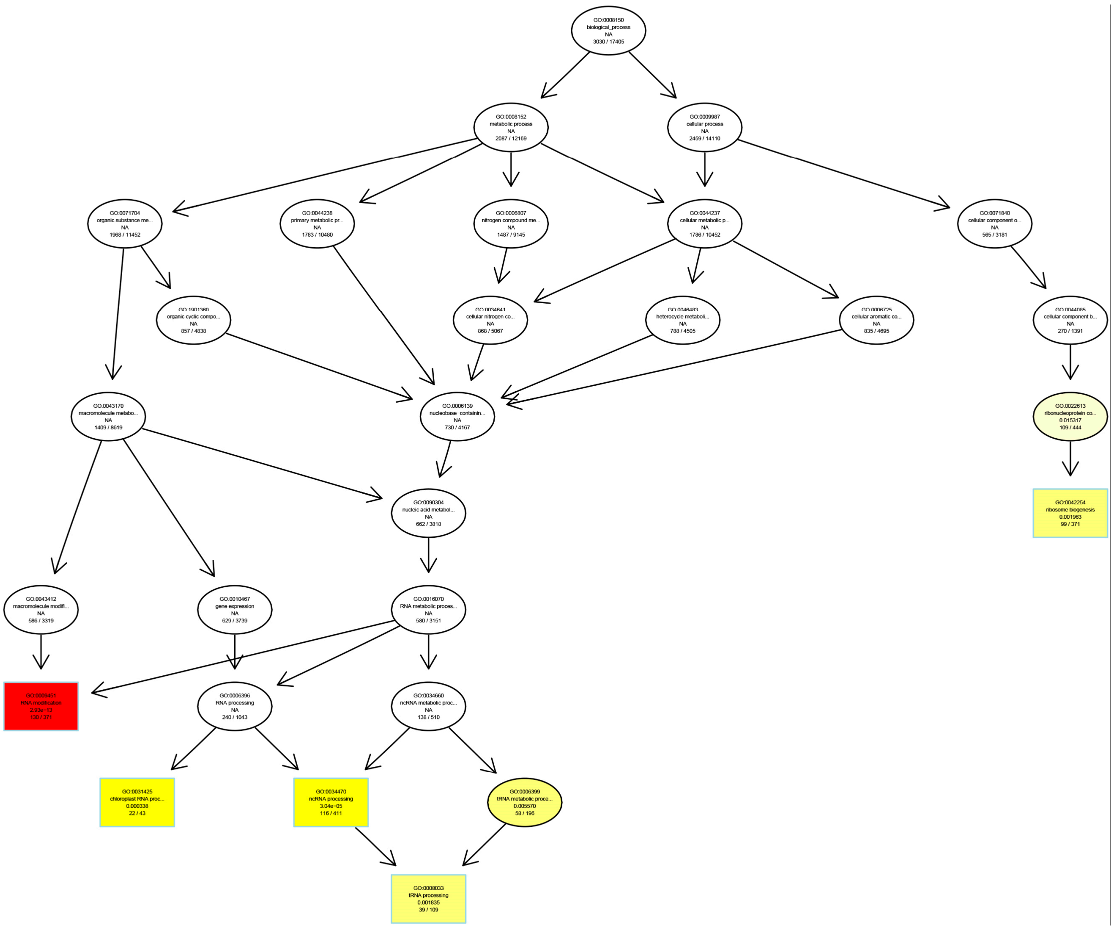

Supplemental Figure S6. The directed acyclic graph for the enriched terms based on biological process-GO. Each node represents a GO entry, the rectangle represents the GO entry of the top 5 enrichment degree selected, and the ellipse represents the contained nodes. The colors of rectangle and ellipse represent the relative enrichment degree. From bright yellow to dark red, the p-value is getting lower and lower, and the significance is getting higher and higher. White represents insignificant. Each node displayed 4 rows of data, representing the ID, function, corrected p-value of the GO entry, the number of differential genes and the total number of genes of the GO entry.

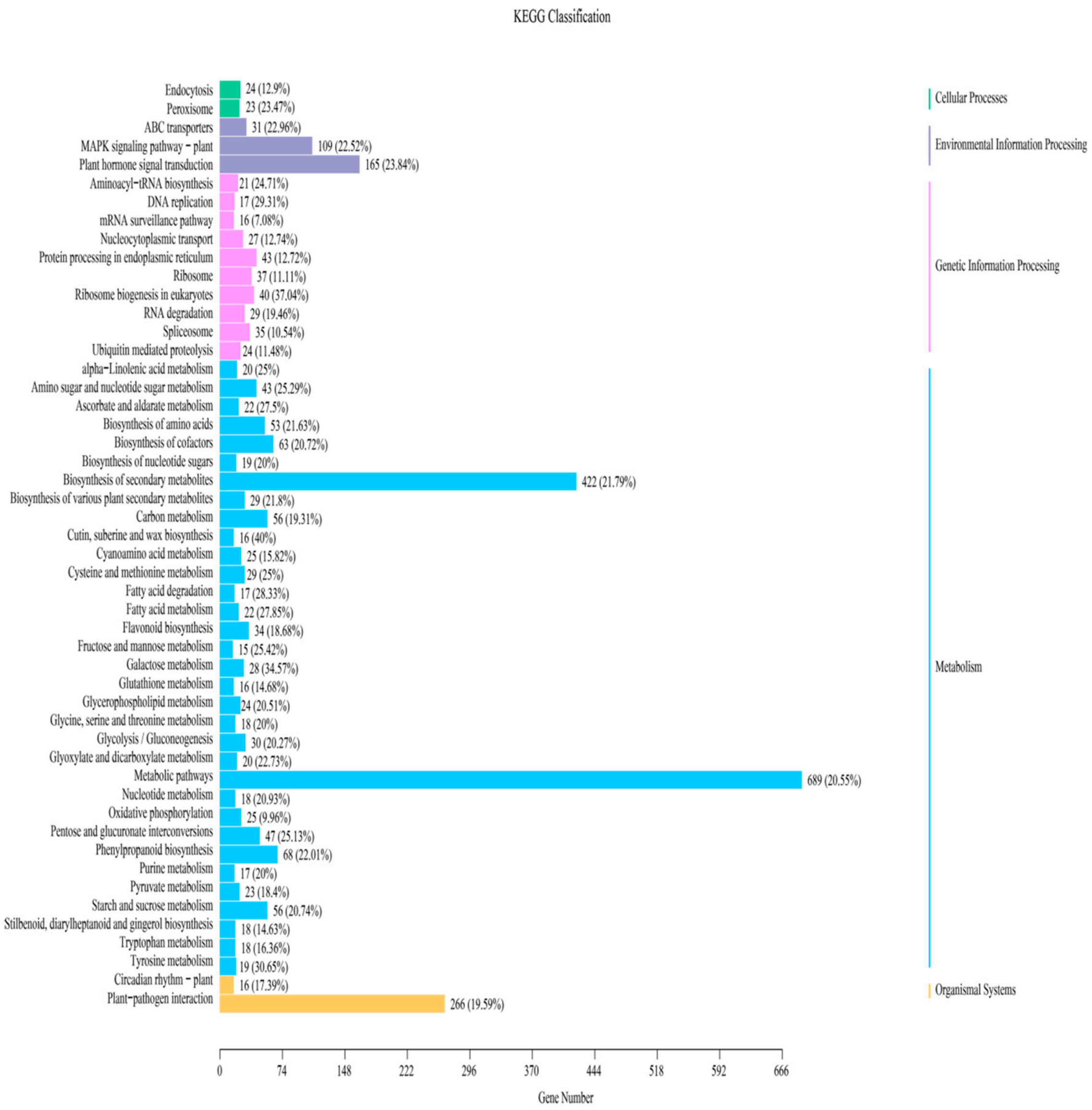

Supplemental Figure S7. KEGG enrichment analysis of DEGs in two *P. mume* cultivars. The abscissa represents the number of differential genes annotated to the pathway, and the ordinate represents the name of the KEGG pathway. The numbers in the figure represent the number of differentially expressed genes annotated to the pathway. The brackets are the specific values of the ratio of the number of differentially expressed genes annotated to the pathway to the number of background genes annotated to the pathway. The rightmost label represents the classification of the KEGG pathway.

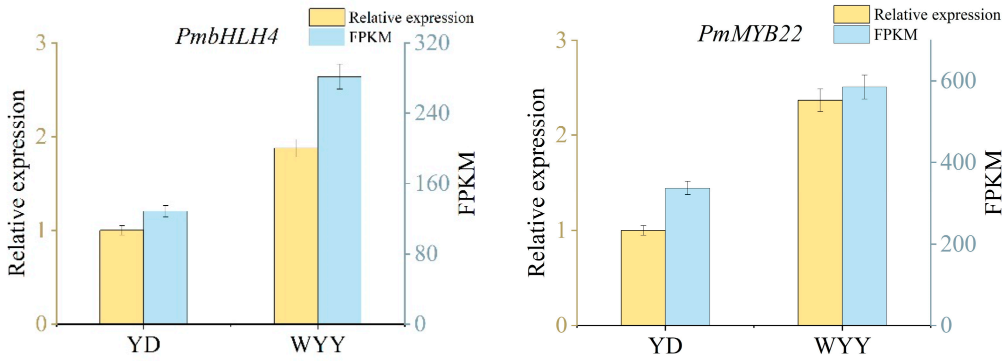

Supplemental Figure S8. RT-qPCR and FPKM of two DEGs identified by RNA sequencing. The y-axis on the left represents the relative gene expression levels ($2^{-\Delta\Delta Ct}$) analyzed by RT-qPCR; the y-axis on the right shows the FPKM value obtained by RNA-seq.
